# Metagenomic and Metabolomic Signatures of Gut Microbiota After *Helicobacter pylori* Eradication Therapy Supplemented with Butyric Acid and Inulin: Observational Study

**DOI:** 10.3390/ijms27198485

**Published:** 2026-09-23

**Authors:** Sayar R. Abdulkhakov, Dilyara D. Safina, Maria I. Markelova, Elizaveta R. Ponomareva, Maria N. Siniagina, Dilyara R. Khusnutdinova, Olga V. Kupriyanova, Rustam A. Abdulkhakov, Tatyana V. Grigoryeva

**Affiliations:** 1 Department of Internal Diseases, Institute of Fundamental Medicine and Biology, Kazan (Volga Region) Federal University, Kazan 420008, Russiaelizavettunina@mail.ru (E.R.P.); dilyahusn@gmail.com (D.R.K.);; 2 Department of Hospital Therapy, Faculty of Medicine, Kazan State Medical University, Kazan 420012, Russia; 3 IQVIA Russia, Moscow 115054, Russia

**Keywords:** gut microbiota, *Helicobacter pylori*, eradication therapy, short-chain fatty acids, butyric acid, inulin, Zacofalk

## Abstract

The long-term impact of eradication therapy for *Helicobacter pylori* on the gut microbiota is a subject of significant interest. To evaluate the influence of the bismuth-based quadruple *H. pylori* eradication therapy supplemented with butyrate and inulin on functional alterations within the gut microbial community, metagenomic and metabolomic profiling were performed. Fecal samples were collected from 15 patients (aged 19–64 years) prior to the treatment, immediately after the treatment, and one month after the therapy. A significant decrease in the relative abundance of Actinobacteria, such as *Bifidobacterium* and *Collinsella*, was observed after the therapeutic intervention. In contrast, the levels of *Faecalibacterium prausnitzii* remained consistent, while *Roseburia faecis* levels exhibited a substantial decline, suggesting a disruption in key butyrate-producing bacteria. A moderate increase in the relative abundance of opportunistic Proteobacteria, Firmicutes, and Bacteroides was observed. A comprehensive analysis of short-chain fatty acids (SCFAs) levels in the feces of the patients revealed a decline in the butyric acid concentration in response to the therapy, followed by a return to baseline levels. However, the level of valeric acid declined, and there was no tendency for it to increase after a month-long course of therapy. It is important to note that these shifts persisted for a period of four weeks. Consequently, the *H. pylori* eradication therapy intervention promotes a persistent microbial reorganization, and the observed recovery is unlikely to be spontaneous and probably reflects the protective effect of butyrate and inulin supplementation. To confirm the conclusions, a larger sample size and longer-duration studies are required.

## 1. Introduction

Eradication of *Helicobacter pylori* is currently indicated in patients presenting with a range of upper gastrointestinal disorders, notably peptic ulcer disease, chronic atrophic gastritis, and gastric mucosa-associated lymphoid tissue (MALT) lymphoma [1,2]. In recent years, however, the clinical rationale has broadened considerably. Current consensus documents—including the Maastricht VI/Florence report, the Kyoto global consensus, the 2024 update of the American College of Gastroenterology guideline, and the recently issued Taipei Global Consensus II—converge on the view that *H. pylori*-associated gastritis is itself an infectious disease. On that basis, treatment is recommended for every infected patient, whether or not symptoms or complications are present, since bacterial clearance resolves the underlying mucosal inflammation and improves gastric outcomes over the long term [1,2,3,4]. The prevalence of *H. pylori* has been declining during the last decades throughout the world, including in Russia, getting down to 42.95% according to a recent systematic review and meta-analysis [5].

The oncogenic potential of the organism further reinforces the case for eradication. *H. pylori* has long been categorized as a Group 1 (definite) human carcinogen by the International Agency for Research on Cancer (IARC), based on robust epidemiological and mechanistic evidence linking chronic infection to non-cardia gastric adenocarcinoma [6,7]. Persistent colonization drives the Correa cascade, in which superficial gastritis progresses to atrophic gastritis, intestinal metaplasia, dysplasia, and ultimately invasive carcinoma [7,8]. Consequently, chronic *H. pylori* gastritis is now regarded as a bona fide precancerous condition [7,9].

Multiple cohort studies, randomized trials, and meta-analyses have shown that eradication lowers the incidence of both primary and metachronous gastric cancer, and a large community-based cluster trial has recently extended this benefit to the population level [9,10,11]. In addition, several studies indicate that microbial clearance can foster partial regression of glandular atrophy in the corpus and antral mucosa, whereas intestinal metaplasia—once fully established—appears to be only marginally reversible and behaves as a largely fixed lesion [8,12]. Taken together, these observations provide a compelling rationale for offering eradication therapy to all *H. pylori*-positive individuals as a strategy for gastric cancer prevention, independent of clinical presentation [1,4,11].

Antimicrobial regimens used to eliminate *H. pylori*—typically combining two or more antibiotics with a proton pump inhibitor—often have a lasting impact on the intestinal ecosystem. Sequencing-based gut microbiota investigations have documented reductions in α-diversity, shifts in taxonomic composition (including expansion of Proteobacteria and contraction of Bacteroidetes, Firmicutes, and Actinobacteria), and functional perturbances of microbial metabolic pathways in the weeks and months following treatment [13,14,15]. These alterations are accompanied by short- and long-term adverse events, ranging from antibiotic-associated diarrhea to enrichment of antimicrobial resistance genes, many of which reflect the intensive pressure of broad-spectrum antibiotics on the commensal microbiota [13,14,16].

Particularly concerning is the depletion of short-chain fatty acid (SCFA)-producing taxa, including key butyrate producers, such as *Faecalibacterium prausnitzii*, *Roseburia* spp., and members of the Lachnospiraceae family [14,17,18]. As known, butyrate and related SCFAs fuel colonocytes, help maintain the integrity of the mucosal barrier, and shape host immune responses. Their depletion is, therefore, thought to explain a considerable share of the metabolic and inflammatory disturbances observed after eradication therapy [17,18,19]. Their high importance in the normal gut has driven interest in adjunctive approaches, such as probiotics, prebiotics, and dietary fiber supplementation, intended to protect microbial function during treatment and to speed recovery of the SCFA-producing community afterwards [16,18,19].

The idea of combining butyric acid with inulin during antibiotic therapy is supported by several complementary mechanisms that have been described recently. These findings suggest that the combination of butyric acid and inulin potentially may not just compensate the temporary loss of microbial butyrate production but also supports the recovery of key bacterial taxa and their metabolic interactions, which are essential for restoring the normal gut function.

As known, exogenous butyric acid provides colonocytes with an immediate energy substrate at a time when microbial butyrate output is depressed, supporting mitochondrial function, tight-junction assembly, and mucosal immunity [20,21].

At the same time, inulin serves as a selective substrate for beneficial members of the microbiota, especially *Bifidobacterium*. Even under antibiotic pressure, these bacteria are able to utilize inulin and contribute to the early stages of recovery. Recent studies have shown that inulin-responsive taxa form a distinct functional group within the gut microbiome, with *Bifidobacteria* playing a central role as primary degraders [22,23].

Inulin selectively supports the growth of *Bifidobacterium* and, indirectly, *F. prausnitzii*, even under antibiotic pressure. Riva et al. (2023) and Sanlier et al. (2026) have recently mapped the inulin-responsive fraction of the human colonic microbiota and confirmed the central role of *Bifidobacterium* as primary degraders [22,23]. Bifidobacterial acetate and lactate produced from inulin fermentation are the substrates that fuel butyrate production by *Roseburia*, *F. prausnitzii*, and *Eubacterium rectale* via the acetyl-CoA/butyryl-CoA CoA-transferase pathway [17,22,23]. The aim of the study was to characterize the short- and long-term functional alterations of gut microbiota after *H. pylori* eradication therapy supplemented with butyrate and inulin based on metagenomic and metabolomic profiles.

## 2. Results

### 2.1. Immediate Taxonomic Changes (Point 1 vs. Point 2)

The administration of standard first-line *H. pylori* eradication therapy resulted in an immediate and significant longstanding restructuration of the gut microbial landscape.

At Point 2 (immediately after the eradication therapy), a dramatic depletion of core *Actinobacteria* was observed, characterized by a near-total loss of the *Bifidobacterium* and *Collinsella* genus (Figure 1).

The relative abundance of *Bifidobacterium adolescentis* decreased significantly from a baseline of 1.88% ± 4.39% (here and throughout, data are presented as mean ± standard deviation) at Point 1 to 0.05% ± 0.02% at Point 2 (*p* = 0.029). Similarly, *Bifidobacterium pseudocatenulatum* exhibited a sharp decline, falling from 2.11% ± 4.09% to 0.22% ± 0.06% (*p* = 0.018). The genus *Collinsella* followed a comparable trajectory, with *Collinsella aerofaciens* (SGB14535) reducing from 1.07% ± 1.17% at baseline to 0.2% ± 0.63% post-therapy (*p* = 0.013).

Key butyrate-producing taxa faced opposite changes: while *F. prausnitzii* remained unchanged, *Roseburia faecis* showed significant reduction, indicating disruption of some butyrate-producing bacteria immediately after the eradication therapy (Table 1).

In contrast to the loss of beneficial commensals, the immediate post-therapy period was marked by an expansion of opportunistic *Proteobacteria* and *Firmicutes*.

Most notably, *Enterococcus faecium* increased (however, not being statistically significant) from 0.30% ± 1.1% at baseline to 6.65% ± 8.54% post-therapy (*p* = 0.186), while *Enterococcus faecalis* (SGB7962) rose from 0.02% ± 0.07% to 2.12% ± 3.96% (*p* = 0.113).

Conversely, *Bacteroides* species showed notable resilience. For example, the relative abundance of *Bacteroides uniformis* showed only a slight increase after antibiotic exposure and did not reach statistical significance compared with baseline levels, suggesting a limited but early post-treatment expansion. This expansion during active antibiotic exposure confirms the intrinsic resistance mechanisms possessed by Bacteroidetes, including multidrug efflux pumps and β-lactamases, and establishes *Bacteroides* as the dominant genus in the immediate post-therapy ecosystem.

The immediate post-therapy period is marked by a profound taxonomic reorganization driven by differential antibiotic susceptibility. *Collinsella* undergoes near extinction-level depletion, while *Bifidobacterium* is substantially reduced, although not completely eliminated.

### 2.2. Longitudinal Taxonomic Changes (Point 1 vs. Point 3)

By four weeks post-therapy (Point 3), partial recovery of the microbiota was observed, although the community did not fully return to baseline composition, suggesting revealing capacity of the gut microbiota. While some species showed signs of resilience, others remained suppressed in the long-term. These changes could be described as the establishment of a new taxonomic equilibrium.

Persistent alterations were evident within the Actinobacteria phylum at Point 3, indicating incomplete community recovery. The depletion of *B. adolescentis* persisted and intensified, with abundance declining to 0.03% ± 0.13% compared with baseline—1.88% ± 4.39% (*p* = 0.018).

In contrast, *B. pseudocatenulatum* showed partial recovery, increasing to 1.29% ± 3.92% (*p* = 0.4). A similar trend was observed for *C. aerofaciens*, which increased to 0.28% ± 0.39% by Point 3, approaching but not fully restoring baseline levels, 1.07% ± 1.19% (*p* = 0.68). These findings suggest partial recovery of select *Actinobacteria*, although full restoration of their metabolic role remains incomplete.

Meanwhile, the transient expansion of *Enterococcus* observed at Point 2 largely resolved by Point 3. *E. faecium* and *E. faecalis* both returned to levels not significantly different from baseline, indicating recovery of colonization resistance (Table 2).

The post-therapy bloom of *Enterococcus* observed at Point 2 largely receded by Point 3, indicating some recovery of community balance. *E. faecium* declined to 0.02% ± 0.06%, while *E. faecalis* decreased to 0.18% ± 0.67%, with neither taxon showing significant deviation from baseline at this later time point.

Among butyrate-producing taxa, *Roseburia faecis* demonstrated stabilization, maintaining levels comparable to baseline—1.33% ± 0.40% at Point 3 vs. 1.32% ± 1.36% at Point 1 (*p* = 0.85). In contrast, *F. prausnitzii* showed a mild increase up to 4.24% ± 3.37%, remaining comparable with baseline, 2.45% ± 1.87% (*p* = 0.077), indicating delayed recovery of this key butyrate-producing species.

Another noticeable taxonomic shift at Point 3 was the further expansion of the Bacteroidetes phylum. While not reaching statistical significance, *Bacteroides uniformis* increased from its baseline abundance—0.43% ± 1.06% to 1.98% ± 3.61% (*p* = 0.1). This expansion likely reflects the occupation of metabolic niches vacated by the collapse of *Actinobacteria*, reinforcing a sustained restructuring of the post-antibiotic gut ecosystem.

### 2.3. Quantitative Overview of Functional Pathway Representation

The analysis revealed significant perturbations in metabolic potential immediately following antibiotic exposure, with a partial functional restoration observed at the four-week period (Figure 2).

The data obtained from the MetaCyc-based analysis reveal a pervasive suppression of *Bifidobacterium*-associated pathways at Point 2, with negligible recovery observed by Point 3. Conversely, pathways associated with *B. uniformis* and other unclassified taxa show significant increases by Point 3, suggesting a permanent or long-term restructuring of the gut metabolic landscape.

### 2.4. Functional Dynamics of Bifidobacterium-Associated Pathways

*B. adolescentis* strains contribute significantly to the functional potential of the baseline microbiota (Point 1). The following analysis details the disruption of these pathways across the three points of the study.

### 2.5. Amino Acid Biosynthesis and Branched-Chain Amino Acid (BCAA)-Related Pathways

Pathways involved in branched-chain amino acid (BCAA) biosynthesis (VALSYN-PWY, ILEUSYN-PWY, BRANCHED-CHAIN-AA-SYN-PWY) showed a marked decline (>95%) at Point 2 (*p* ≈ 0.02), with no recovery by Point 3. BCAAs, including L-valine and L-isoleucine, are essential for host protein synthesis and the regulation of insulin sensitivity. The MetaCyc pathways VALSYN-PWY and ILEUSYN-PWY represent the core of this biosynthetic potential.

As shown in Table 3, the average abundance of valine and isoleucine biosynthesis pathways decreases by more than 95% at Point 2. This functional collapse is significant (*p* < 0.05) and persists through Point 3, with pathway activity remaining at near-minimal levels.

### 2.6. Nucleotide Biosynthesis and Salvage Pathways

Microbial nucleotide synthesis is critical for bacterial proliferation and community stability. The PWY-6123 (inosine-5′-phosphate biosynthesis II) and PWY-7221 (guanosine ribonucleotides de novo biosynthesis) pathways are primary indicators of this potential.

As is known, biosynthesis of purines, particularly guanosine ribonucleotides, requires a large amount of energy. At Point 1, these pathways are highly active, 15.32 cpm ± 38.74 cpm (cpm—counts per million mapped reads), indicating strong metabolic capacity (Table 4). In contrast, their levels drop sharply at Point 2, 0.29 cpm ± 1.15 cpm, and Point 3, 0.11 cpm ± 0.43 cpm, where they are almost absent. These data suggest that the microbial community has a much lower ability to support fast cell growth and maintain DNA and RNA through repair and replication at later points while their role in this process remains prevalent and crucial, suggesting decreased microbial growth potential, particularly within *Actinobacteria*.

### 2.7. Energy Metabolism and Fermentation Shunts Pathways

The pyruvate fermentation pathway (PWY-7111), which converts pyruvate into isobutanol, and the Rubisco shunt (PWY-5723) represent alternative ways for microbes to redirect carbon flow. PWY-7111 shows the same sharp and sustained decline observed for the amino acid biosynthesis pathways, suggesting that this metabolic option is largely lost after the initial time point (Table 5).

Interestingly, the Rubisco shunt behaves very differently. It increases dramatically at Point 2, 122.77 cpm ± 147.82 cpm, compared with Point 1, 3.50 cpm ± 9.44 cpm. It can be suggested that the transient surge likely reflects a stress-induced shift in carbon fixation potential with certain bacteria, which can be temporally enriched immediately after the completion of eradication therapy, potentially antibiotic-tolerant or resistant populations that can rapidly upregulate this pathway, before returning toward a lower abundance at Point 3. By Point 3, activity in this pathway declines again, 11.57 cpm ± 17.34 cpm, indicating that this shift is short-lived rather than a stable feature of the community, consistent with the sustained expansion of *Bacteroides* and suppression of *Collinsella* and *Bifidobacterium*.

In the gut microbiome context, this pathway could reflect alternative carbon utilization routes linked to pentose phosphate metabolism and downstream propionate production. This shift suggests a redistribution of carbon flow within the microbial community rather than true carbon fixation.

### 2.8. Analysis of Collinsella-Associated Structural and Metabolic Pathways

*C. aerofaciens* is another vital contributor to the healthy gut’s structural integrity and inflammatory regulation. Its functional potential is largely related to peptidoglycan biosynthesis and the methylerythritol phosphate pathway.

### 2.9. Peptidoglycan Biosynthesis and Immunomodulation-Related Pathways

Peptidoglycan synthesis is essential for bacterial cell wall formation. In addition, peptidoglycan fragments act as key immunological signals by engaging intracellular pattern-recognition receptors, such as NOD1 and NOD2, thereby contributing to the regulation of innate immune responses.

At Point 1, the peptidoglycan biosynthesis pathway (PEPTIDOGLYCANSYN-PWY) is strongly represented—10.31 cpm ± 11.63 cpm. By Point 2, its abundance drops sharply—1.29% ± 5.005%, *p* = 0.024—indicating a major reduction in the production of these immune-active bacterial components (Table 6).

Unlike the *Bifidobacterium* pathways, *Collinsella*’s peptidoglycan pathways show a statistically significant but mild recovery trend at Point 3—2.19 cpm ± 3.907 cpm, *p* = 0.048. However, this recovery is only partial, as the Point 3 abundance remains less than 25% of the baseline value.

A nearly identical pattern was observed for PWY-6385 (peptidoglycan biosynthesis III), which decreased from 10.06 cpm ± 11.40 cpm at Point 1 to 1.03 cpm ± 4.03 cpm at Point 2 (*p* = 0.024), with partial recovery to 2.21 cpm ± 3.92 cpm at Point 3 (*p* = 0.04). The parallel dynamics of these two distinct peptidoglycan synthesis pathways suggest a global suppression of *Collinsella* cell wall biosynthesis capacity rather than pathway-specific regulation.

HISTSYN-PWY (L-histidine biosynthesis), while not directly involved in peptidoglycan formation, followed the same temporal pattern, declining from 6.25% ± 7.2% at baseline to 0.36 cpm ± 1.4 cpm immediately post-therapy, and recovering to only 1.41 cpm ± 3.13 cpm by Point 3.

### 2.10. Terpenoid Biosynthesis and the Methylerythritol Phosphate Pathway

The NONMEVIPP-PWY (methylerythritol phosphate pathway) is used by most bacteria to synthesize the precursors of isoprenoids, which are vital for cell membrane stability and electron transport. The relative abundance of this pathway decreases from 7.05 cpm ± 7.87 cpm at Point 1 to 0.92 cpm ± 3.58 cpm at Point 2 and continues to decline to 0.35 cpm ± 1.37 cpm at Point 3 (Table 7). This persistent decline (*p* = 0.031) suggests a sustained impairment of isoprenoid-related cellular functions within the Actinobacterial community members, which has long-term implications for the community’s structural resilience.

### 2.11. Functional Reorganization Toward Bacteroides-Dominated Pathways

While the functional potential of *Bifidobacterium* and *Collinsella* declines, the functional activity of *Bacteroides* members of the gut community has been expanding, suggesting a shift in community roles and their taxonomic abundance—*B. uniformis* and several *Blautia*, in particular.

Pathways involved in carbohydrate utilization and cell wall biosynthesis (e.g., dTDP-L-rhamnose biosynthesis and UDP-N-acetylmuramoyl-pentapeptide biosynthesis) increased by Point 3 (Table 8). This pattern suggests functional replacement, where *Bacteroides* taxa occupy metabolic niches vacated by Actinobacteria.

### 2.12. Carbohydrate and Sugar-Nucleotide Pathways

The dTDP-L-rhamnose biosynthesis pathway (DTDPRHAMSYN-PWY: dTDP-L-rhamnose biosynthesis) is required for producing rhamnose-containing cell wall polysaccharides, which are important for bacterial survival and structural integrity. As shown in Table 8, the abundance of this pathway increases slightly from 3.36 cpm ± 9.29 cpm at Point 1 to 15.66 cpm ± 25.06 cpm by Point 3, *p* = 0.050.

PWY-6387: UDP-N-acetylmuramoyl-pentapeptide biosynthesis I (meso-diaminopimelate-containing), a key component of peptidoglycan production in *Bacteroides,* and PWY-6123 (Inosine-5′-P II), do not show significant changes from Point 1 to Point 3 (*p* > 0.05 for both pathways).

Together, these changes support a clear functional replacement process: as metabolic functions associated with *Bifidobacterium* decline, *Bacteroides* expands its contribution to cell wall biosynthesis, effectively occupying metabolic niches left vacant within the community.

Overall, these data illustrate a functional replacement phenomenon in which *Bacteroides*-driven pathways increase in prominence as *Bifidobacterium*-associated functional potential is lost, leading to a shift in the taxa most actively supporting core cell wall biosynthetic functions.

### 2.13. Folate and Vitamin Metabolism

Folate transformation pathways (PWY-3841: folate transformations II) are critical for one-carbon metabolism and energy production. Analysis of vitamin metabolism pathways revealed a delayed enrichment in folate and pantothenate biosynthetic capacity by four weeks post-eradication therapy; however, it was statistically non-significant, with no changes immediately after completing eradication therapy (Table 9).

The relative abundance of pantothenate and folate biosynthesis pathways in *Bacteroides* increases as well by Point 3; however, it does not reach a statistically significant difference. These data suggest that the recovery phase (Point 3) is characterized mainly by a “*Bacteroides*-dominant” metabolic state, rather than a return to the original metabolic condition of the gut microbiota, which likely reflects meaningful differences in metabolic efficiency and the way microbiota interacts with the host.

### 2.14. Altered Histidine and Arginine Flux

Histidine biosynthesis (HISTSYN-PWY) is effectively lost within the *Collinsella* niche, while both histidine degradation (HISDEG-PWY) and histidine biosynthesis within the *Bacteroides* niche remain unchanged at Point 2 with a tendency to increase by four weeks post-therapy (Table 10). Together, these opposing trends suggest a net reduction in the availability of L-histidine in the gut lumen. Given histidine’s role in proton buffering reactive oxygen species, this metabolic shift may render the gut environment more susceptible to oxidative stress and inflammatory fluctuations.

The same tendency was noticed in arginine biosynthesis (ARGININE-SYN4-PWY) by *Bacteroides uniformis*, which may represent a compensatory response; however, it did not reach statistical significance. Arginine serves as a key nitrogen source and a precursor for nitric oxide, a molecule with well-established antimicrobial and immunomodulatory functions, suggesting an adaptive reconfiguration of nitrogen and redox metabolism in the post-therapy gut ecosystem.

### 2.15. Analysis of Functional Capacity Regarding Short-Chain Fatty Acids (SCFAs)

The absolute level of butyric acid decreased significantly from 0.607 mg/g at Point 1 to 0.226 mg/g immediately after eradication therapy (p_1–2_ = 0.004), with a further increase to 0.491 mg/g (p_2–3_ = 0.012) by Point 3 and being comparable by four weeks after eradication therapy with the initial level (p_1–3_ = 0.094). However, the level of valeric acid decreased from 0.146 mg/g initially to 0.048 mg/g at Point 2 (p_1–2_ = 0.007) and remained lower than the initial level four weeks after the end of eradication therapy—0.092 mg/g (p_1–3_ = 0.007; Figure 3).

The dynamics of SCFA levels correspond with the changes related to SCFA-related metabolic pathways.

Based on a metagenomic analysis of the provided data, the functional capacity of the gut microbiota regarding SCFA metabolism undergoes a profound disruption following eradication therapy. SCFAs, primarily acetate, propionate, and butyrate, are critical metabolites for maintaining intestinal barrier integrity, regulating host immunity, and preventing the overgrowth of pathogenic species.

### 2.16. Suppression of Primary Fermentative Pathways

The most significant impact of the therapy is the near-total collapse of pathways associated with *Bifidobacterium* and *C. aerofaciens,* which are primary producers of acetate and lactate in the healthy gut.

One of the key changes can be described as loss of primary fermentation capacity. Pathways associated with acetate and lactate production, particularly the Bifidobacterium shunt (P124-PWY), were significantly reduced at Point 2 and remained below baseline at Point 3.

The Bifidobacterium shunt (P124-PWY): Due to being the central metabolic pathway in *Bifidobacteria* it is vital for converting hexoses into acetate and lactate. The results show a sharp decline from 93.47 cpm ± 92.31 cpm at Point 1 to 25.71 cpm ± 44.42 cpm at Point 2 (*p* = 0.01), with only partial recovery to 38.89 cpm ± 71.39 cpm at Point 3, but still significantly lower than it was at baseline (*p* = 0.036).

As known, acetate and lactate serve as substrates for butyrate-producing bacteria, so this reduction likely limits cross-feeding interactions within the community. The functional absence of Bifidobacterial acetate producers likely explains the decrease of absolute butyrate production at Point 2 and only partial restoration by Point 3, remaining below the baseline.

### 2.17. Depletion of Precursors for Butyrate Production

Butyrate is the preferred energy source for colonocytes and is essential for preventing “leaky gut” and inflammation. The data of the study show a significant loss of “primary polysaccharide degradation” capacity, which is the step for butyrate production.

Sucrose degradation (PWY-5384): Sucrose degradation capacity in *Bifidobacterium* has almost disappeared from Point 1, 11.96 cpm ± 28.48 cpm, to Point 2, 0.33 cpm ± 1.3 cpm, *p* = 0.045, with no recovery at Point 3, 0.15 cpm ± 0.6 cpm, compared to Point 1 (*p* = 0.043). The loss of these primary degraders creates a substrate deficit for the broader community, likely explaining why overall butyrate-producing bacteria levels often remain depressed following antibiotic therapy.

*Blautia* glycolysis: There is a stable state in ANAGLYCOLYSIS-PWY: glycolysis III (from glucose), associated with *Blautia* sp., not changing from Point 1, 9.73 cpm ± 12.52 cpm, to Point 3, 10.74 cpm ± 25.41 cpm, *p* = 0.86. *Blautia* contributes to acetate production; however, it is likely that the value of this stable state is insufficient to offset the loss of the *Bifidobacterium* shunt due to its limited capacity.

*Bacteroides* expansion: *B. uniformis* expands its representation in carbohydrate degradation pathways (DTDPRHAMSYN-PWY: dTDP-L-rhamnose biosynthesis) from Point 1 to Point 3, increasing significantly from 3.36 cpm ± 9.29 cpm to 15.67 cpm ± 25.06 cpm, *p* = 0.05. As is known, *Bacteroidetes* are primary propionate producers via the succinate pathway and acetate rather than butyrate. This shift would increase propionate production capacity while potentially reducing butyrate production, fundamentally altering the SCFA profile.

Glycan degradation capacity: The stable state of complex carbohydrate degradation pathways at Point 3 was noted for (GLCMANNANAUT-PWY: superpathway of N-acetylglucosamine, N-acetylmannosamine, and N-acetylneuraminate) degradation pathway abundance, which remains stable from Point 1, 8.57 cpm ± 12.25 cpm, to Point 3, 11.63 cpm ± 39.94 cpm, *p* = 0.14. However, controversially, the relative abundance of the pathway drops significantly immediately after the therapy (Point 2—1.45 cpm ± 2.36 cpm, *p* = 0.02).

The relative abundance of the pathway PWY-6906: chitin derivatives degradation has been continually increasing from Point 1, 5.32 cpm ± 10.80 cpm, reaching a maximum at Point 3—22.56 cpm ± 27.23 cpm, *p* = 0.03. These data may indicate the enhanced capacity of microbiota to utilize host-derived glycans and dietary fibers as an acute stress response (immediate post-therapy). This expanded glycan-degrading capability could increase overall SCFA substrate availability, potentially compensating for the reduced butyrogenic capacity.

Butyrate, the primary energy source for colonocytes, is produced either from acetate via the butyryl-CoA:acetyl-CoA transferase pathway or from lysine through the glutarate pathway. The marked reduction in L-lysine biosynthesis (DAPLYSINESYN-PWY) associated with *B. adolescentis*—declining from 12.88 cpm ± 31.27 cpm at baseline to 0.33 cpm ± 1.27 cpm at Point 2 (*p* = 0.05), with a further decrease to 0.15 cpm ± 0.58 cpm at Point 3 (*p* = 0.05)—indicates a sustained loss of lysine production.

Because lysine serves as a key precursor that competes with glutarate for butyrate synthesis, this prolonged deficit may constrain butyrate production. This metabolic limitation provides a plausible explanation for the incomplete recovery of butyrate-producing pathways, despite the apparent restoration of overall microbial diversity at later time points.

### 2.18. Correlation Between Species and SCFAs

*Bifidobacterium pseudocatenulatum* and *Faecalibacterium prausnitzii* show a moderate positive correlation with the concentration of butyric acid in the samples (0.32, *p* = 0.033, and 0.36, *p* = 0.017, respectively), while *Enterococcus faecium* shows a moderate negative correlation (−0.42, *p* = 0.006; Table 11). *Faecalibacterium prausnitzii* correlates positively with valeric acid (0.37, *p* = 0.013), while *Bacteroides uniformis* correlates negatively (−0.39, *p* = 0.009).

## 3. Discussion

The present study provides a detailed view of the gut microbiota response to *H. pylori* eradication therapy when combined with butyric acid and inulin supplementation. Our results confirm several well-established patterns: depletion of Actinobacteria, transient increase of *Enterococcus*, and expansion of *Bacteroides*, while also favorable ecological resilience, which might also be consistent with a protective effect of the butyric acid + inulin combination [24,25].

One of the most consistent findings in our data is the marked depletion of *Actinobacteria*, particularly *Bifidobacterium* and *Collinsella*, immediately after therapy. This pattern aligns closely with earlier metagenomic observations, including those reported by Olekhnovich et al. (2019) [25], and has since been confirmed in multiple studies examining the microbiome response to eradication regimens. Subsequent independent cohorts and a recent systematic review have confirmed that Actinobacteriota depletion is one of the most reproducible and clinically relevant hallmarks of any macrolide-containing eradication regimen, largely independent of antibiotic combination or geographic setting [26,27].

The persistence of *B. adolescentis* suppression at four weeks in our study is, therefore, expected. However, the partial recovery of *B. pseudocatenulatum* and of *Collinsella* by Point 3 is more encouraging and is one of several observations that differ from the pattern reported in the untreated arm of our previous comparison study [24].

Because *B. adolescentis* is a highly efficient inulin fermenter and one of the principal acetate producers in the healthy human colon, its slow recovery has potential metabolic consequences. Riva et al. (2023) recently identified *B. adolescentis* among the most consistently inulin-responsive members of the human colonic microbiota [22], and Rivière et al. (2016) demonstrated that acetate generated by *B. adolescentis* is directly cross-fed to *F. prausnitzii* to drive butyrate synthesis via the acetyl-CoA/butyryl-CoA CoA-transferase pathway [17]. This connection is a plausible link between the observed collapse of the *Bifidobacterium* shunt (P124-PWY) and the reduced butyrate-producing potential detected in the functional profile, despite the absence of strong suppression of *F. prausnitzii*. The reduction of butyrate-producing capacity corresponds with the statistically significant decrease of absolute levels of SCFAs, particularly butyrate, immediately after eradication therapy, with a mild increase four weeks after the end of therapy, being comparable with the initial level.

In this context, the absence of marked suppression of *F. prausnitzii* observed in our study is of particular importance. This species is a major colonic butyrate producer and a central source of anti-inflammatory signals, both through butyrate itself and through the microbial anti-inflammatory potential [28,29]. Its reduced abundance after antibiotic exposure has been repeatedly associated with dysbiosis and impaired mucosal homeostasis [28]. In our cohort, maintaining *F. prausnitzii* almost at the initial level may be partly explained by the supplementation of eradication therapy with butyric acid + inulin, despite both direct antibiotic pressure and the loss of cross-feeding substrates from *Bifidobacterium* and *Collinsella*, which was described and deeply explored by Fishbein et al. in their review [30].

Considering these taxa to function as keystone members of metabolic cross-feeding networks that support butyrate production, their loss is likely to cause functional disruption of carbohydrate fermentation and downstream metabolite exchange. In particular, the depletion of primary starch- and fiber-degrading bacteria may impair metabolic cross-feeding pathways essential for maintaining short-chain fatty acid homeostasis.

In contrast, not all butyrate-producing taxa behaved in the same way. One of the most notable findings of our study is the full recovery of *R. faecis* and several other *Firmicutes* by the four-week time point. This is an important observation, as *Roseburia* species are among the most common producers of butyrate in the healthy human colon and are often considered key markers of a well-functioning and balanced gut ecosystem [20,31]. Their recovery suggests that, despite the initial disruption, parts of the butyrate-producing network remain resilient. This finding differs from earlier reports where recovery of butyrate producers was slower or incomplete and may reflect the influence of the butyric acid + inulin supplementation used in this study.

In the cohort described by Olekhnovich et al. (2019), where eradication therapy was given without any microbiota-supporting intervention, *Roseburia* did not fully recover within the follow-up period, and the overall community remained functionally depleted in terms of butyrate production [25]. In contrast, our previous randomized study showed that patients receiving butyric acid and inulin maintained a significantly higher abundance of the key butyrate-producing pathway (CENTFERM-PWY), while this pathway remained depressed in the standard therapy group [24]. Taken together, these findings suggest that the recovery we observe is unlikely to be purely spontaneous and may instead reflect a protective effect of the supplementation on the butyrate-producing community.

This interpretation is further supported by our metabolomic data. In the same clinical setting, we observed that the decrease in fecal butyric, isovaleric, and valeric acids after therapy was less pronounced in patients receiving Zacofalk compared to those on standard treatment [32]. This is an important distinction, because metagenomic pathway abundance reflects only the potential of the microbiota to produce certain metabolites. In this study, after the butyrate + inulin treatment, a decrease in SCFA levels was observed, although there was a trend toward recovery. The levels of valeric acid decreased at Point 2 and did not tend to increase until the end of the therapy. This phenomenon is probably due to the demise of key SCFA producers, such as *F. prausnitzii, B. adolescentis, B. pseudocatenulatum,* and *R. faecis* [33,34]. The correlation of *B. pseudocatenulatum* may be related to the phenomenon of cross-feeding due to the supply of *F. prausnitzii* acetate as a substrate for the synthesis of butyrate. At the same time, the gut microbiota shifts toward the utilization of substrates rather than their synthesis. This is supported by a positive correlation between the proportion of several bacterial species and the SCFA concentrations. A negative correlation with the SCFAs was observed for *E. faecium* and *B. uniformis. E. faecium* has been associated with the colonic inflammation in patients with ulcerative colitis [35]. This may be explained by the ability of a mixture of SCFAs to inhibit the growth of *E. faecium*, which was shown by King et al. [36]. In our case, a decrease in the concentration of SCFAs led to an increase in the number of *E. faecium*. The bacteria *Bacteroides uniformis* is part of the normal gut microbiota involved in the utilization of polysaccharides [37]. Its negative correlation is probably related to the increase in the number of species, which may be due to the addition of inulin in the preparation [38]. Various strains of *B*. *uniformis* are potential probiotics. They are associated with suppressing inflammation and reducing cases of colitis [39], which can have a positive effect on the condition of patients during therapy and subsequent recovery. The fact that the functional signals we observe are mirrored by real changes in SCFA levels strengthens the argument that these effects are biologically meaningful rather than purely theoretical.

General changes in the representation of microorganisms and the content of SCFAs in the samples demonstrate a different recovery rate. The taxonomic profile is generally restored earlier than the metabolomic profile. Thus, we can see the need for a longer restoration of the functional potential associated with the synthesis of SCFAs.

The increase in *E. faecium* and *E. faecalis* abundance immediately after therapy (however, not reaching statistical significance) was not unexpected. A similar pattern has been described many times in the context of antibiotic exposure, and it fits closely with the classical model of antibiotic-induced loss of colonization resistance, in which niches vacated by primary fermenters are transiently occupied by anaerobes and opportunistic Gram-positive cocci [30,40]. The same observation was reported by Olekhnovich et al. (2019) following *H. pylori* eradication therapy, and more recent work has linked this shift to an increase in antibiotic resistance genes during the post-treatment period [25,27].

The fact that both enterococcal species returned to values statistically indistinguishable from baseline within four weeks in our cohort suggests that colonization resistance was restored relatively quickly. Interestingly, this pattern is consistent with the reduced accumulation of antibiotic-resistance genes previously observed in the Zacofalk-supplemented arm of our randomized comparison [24].

By the four-week time point, we observed a stable abundance of *B. uniformis*, along with a rise in several pathways associated with *Bacteroides*, including the dTDP-L-rhamnose biosynthesis, and pathways linked to succinate and propionate production. Taken together, this suggests that as *Actinobacteria* decline after therapy, *Bacteroides* begin to occupy the available ecological niche, taking a prevailing role in the metabolic workload.

However, some consideration should be taken regrading this pattern. Recent work by Wang X. et al. (2024) showed that *Bacteroides*-derived propionate can support, by producing via methylmalonyl-CoA mutase, goblet cell function and mucus production, suggesting that this shift is not necessarily harmful [41].

This likely represents a partial functional compensation. As butyrate-producing pathways are disrupted, the microbiota appears to partially compensate by increasing propionate-related activity. At the same time, this compensation is not equivalent. Butyrate remains the main energy source for colonocytes and plays a particularly important role in immune regulation, especially in the induction of regulatory T cells [21,42]. While the *Bacteroides* may help maintain some metabolic functions, they likely do not fully replace the role of the original butyrate-producing community.

So, the pronounced loss of beneficial commensals facilitates the expansion of *Enterococcus*, a genus commonly associated with antibiotic-associated diarrhea and hospital-acquired infections. In contrast, *Bacteroides* increases its relative dominance, likely reflecting its intrinsic resilience to antibiotic exposure. Together, these selective pressures give rise to a fundamentally altered microbial community structure with immediate consequences for metabolic network integrity, particularly affecting lactate-producing populations, such as *Collinsella* and *Bifidobacterium*, and potentially reshaping downstream butyrate synthesis.

This new taxonomic equilibrium characterized by reduced *Actinobacteria* (*Bifidobacterium*) and expanded *Bacteroidetes* provides a structural basis for the functional metabolic remodeling observed in parallel pathway analyses, particularly the shift from butyrogenesis toward propionogenesis.

In contrast, the failure of *B. adolescentis* to recover by Point 3 may impose a lasting metabolic scar on the gut ecosystem. Given that *B. adolescentis* is a major starch degrader and acetate producer, its sustained depletion may have long-term consequences for the host SCFA pool, particularly acetate and butyrate levels that are essential for metabolic regulation and immune homeostasis. In this setting, maintaining the abundance of *F. prausnitzii*, a key butyrate producer, is of exceptional importance.

These findings suggest that standard eradication therapy may induce a persistent regime shift in the gut ecosystem unless recovery is supported by targeted microbiota-directed interventions, such as precision probiotics or dietary modulation.

### Summary of Functional Changes

The most clinically meaningful functional shifts in our data were related to short-chain fatty acid metabolism. These changes appear early after therapy and remain only partially resolved at four weeks, suggesting that this aspect of microbiota function is particularly sensitive to antibiotic exposure.

One of the first effects we observed was a clear reduction in pathways responsible for acetate and lactate production, including the Bifidobacterium shunt (P124-PWY) and pyruvate fermentation routes. This is consistent with the loss of primary fermenters, such as *Bifidobacterium* and *Collinsella*. Since *Collinsella* species are primary lactate producers that feed lactate-utilizing butyrogens (*Eubacterium* and *Anaerositpes*), this decline has direct implications for SCFA metabolism, creating a long-lasting limitation in the lactate-to-butyrate cross-feeding network and thus likely impairing butyrate production. Considering acetate and lactate as key substrates for butyrate-producing bacteria, their depletion likely disrupts the normal cross-feeding interactions within the microbial community.

*Collinsella*-associated peptidoglycan biosynthesis is almost completely lost immediately after antibiotic treatment, highlighting the high sensitivity of this genus to the antibiotic therapy. This reduction is likely to affect normal immune signaling at the mucosal surface, as peptidoglycan fragments from commensal bacteria may act as key signals for host pattern-recognition receptors, such as NOD-like receptors. Even four weeks later, recovery remains limited, indicating prolonged functional impairment. It could be suggested that this sustained suppression of *Collinsella*-related metabolic activity may lead to reduced peptidoglycan production affecting innate immune stimulation and consequently weakening epithelial barrier support, with potential consequences for host immune homeostasis.

When looking at butyrate production itself, the picture is more complex. Some butyrate-producing taxa showed encouraging signs of recovery—in particular, *R. faecis* returned to baseline levels by the four-week time point—while *F. prausnitzii* remained almost unchanged throughout the study. Despite the fact that part of the butyrate-producing network is recovering, it is not fully restored. In addition, the reduced availability of upstream substrates means that even with taxonomic recovery, overall butyrate production capacity may still be limited.

At the same time, we observed a shift in the opposite direction, with increasing activity of pathways linked to propionate production. This appears to be driven by the maintenance of *Bacteroides*, particularly *B. uniformis*, and enhanced carbohydrate degradation. Taken together, these findings point to a possible gradual transition from a butyrate-dominated to a more propionate-oriented metabolic profile.

Beyond the SCFA-related pathways, we also saw noticeable changes in several other core metabolic processes. In particular, pathways involved in branched-chain amino acid (BCAA) synthesis, purine metabolism, cell wall components, such as peptidoglycan, and polyamine and S-adenosylmethionine (SAM) biosynthesis were all clearly affected. Most of these functions were largely linked to *Bifidobacterium* and *Collinsella*, which helps explain why their depletion had such a broad functional impact.

This pattern is entirely consistent with the functional findings of our earlier study, where the standard eradication regimen led to substantially more pathway-level perturbations compared to the regimen supplemented with butyric acid and inulin [24]. The persistence of BCAA-biosynthetic depression at Point 3 corresponds with the incomplete recovery of *B. adolescentis*.

## 4. Materials and Methods

### 4.1. Study Design

This prospective study was approved by the Local Ethics Committee of Kazan Federal University (Protocol No. 1, dated 23 February 2015). All included subjects who were patients of the Kazan Federal University Hospital outpatient gastroenterology unit and Kazan State Medical University outpatient clinic (Kazan, Russian Federation) signed written informed consent forms.

Patients were included into the study according to the inclusion/exclusion criteria published previously [24]. The C^13^-urea breath test was used for *H. pylori* detection initially and 4–6 weeks after the end of eradication therapy.

A total of 15 *H. pylori*-positive eligible patients aged 19 to 64 years were enrolled in the study.

Indications for *H. pylori* eradication in the enrolled patients were the following: non-investigated dyspepsia (*n* = 7, 46.7%), suspected long-term PPI use (*n* = 4, 26.7%), and histologically proven atrophic gastritis (*n* = 4, 26.7%).

According to the national guidelines, all subjects received the bismuth-based quadruple *H. pylori* eradication therapy adjusted with butyric acid + inulin supplement: esomeprazole 20 mg, clarithromycin 500 mg, amoxicillin 1000 mg, bismuthate tripotassium dicitrate 240 mg, twice daily, and butyric acid + inulin (Zacofalk) taken in two tablets (one tablet of butyric acid + inulin corresponds to 250 mg of butyric acid and 250 mg of inulin, doses used according to the instruction of the medication), daily, per os, for 14 days.

Fecal samples were collected from each subject at three time points, as follows:

(1) Prior to the beginning of eradication therapy to evaluate the baseline composition of the gut microbiota (Time Point 1).

(2) After the end of eradication therapy (within 14 (+3) days from the beginning of eradication therapy) to evaluate the influence of the eradication therapy on the gut microbiota (Time Point 2).

(3) Within a month (+5 days) of the end of eradication therapy to evaluate the long-term effects of *H. pylori* eradication on the gut microbiota (Time Point 3).

The final study population consisted of 15 *H. pylori*-positive patients who were treated with the abovementioned *H. pylori* first-line eradication therapy. No refusal or loss of follow-up were reported in this study.

Fecal samples were collected in disposable plastic containers. Samples were frozen on the same day and then stored at −80 °C until metagenomic analysis was performed.

### 4.2. Metagenomic Sequencing and Bioinformatic Analysis

The isolation of DNA from fecal samples was accomplished by employing the FastDNA Spin Kit for Faeces (MP Biomedicals, Santa Ana, CA, USA). Library preparation was performed using the NEBNext Ultra II DNA Library Prep Kit for Illumina (New England Biolabs, Ipswich, MA, USA). Quality control was conducted on a 2100 Bioanalyzer (Agilent Technologies, Santa Clara, CA, USA). Subsequent shotgun sequencing was carried out on the NextSeq 500 and HiSeq 1000 platforms (Illumina, San Die-go, CA, USA).

Raw genomic data underwent initial preprocessing with FastQC 0.12, which included low-quality read filtering according to the method described before [43]. The filtered reads were mapped to the human reference genome (UCSC hg19) using Bowtie 2 [44]. Reads that did not map to the human genome were subsequently analyzed with the MetaPhlAn version 4.0 software package [45] to assess the taxonomic diversity of the community. The taxonomic composition of the gut microbiota was characterized at species level. Functional annotation of non-human sequencing reads was performed with HUMAnN3 [46] against the MetaCyc database. Gene representation of metabolic pathways is presented as cpm—counts per million mapped reads.

### 4.3. GC Analysis of SCFA

Sample solution preparation: Fecal samples were collected in falcon tubes and stored at −80 °C. Just before the analysis, the sample was thawed, weighted (between 0.5–1.0 g), and added to a sodium bicarbonate 10 mM solution (1:1 *w*/*v*). The obtained suspension was stirred in a vortex apparatus, extracted in an ultrasonic bath (for 5 min), and then centrifuged at 5000 rpm (for 10 min). An aliquot of 100 μL of the supernatant (corresponding to 0.1 mg of the fecal sample) was added to 1 mL of tert-butyl methyl ether and 50 μL of 1.0 M HCl solution. Afterwards, the tube was shaken in a vortex apparatus for 2 min, centrifuged at 10,000 rpm for 5 min, and finally the solvent layer was transferred in an autosampler vial and analyzed by the GC method.

GC analysis: The SCFA analysis was performed by a gas chromatograph “Clarus 680” (Perkin Elmer, Waltham, MA, USA) with a flame ionization detector (GC-FID). The injection was carried out using an autosampler with a microsyringe, “Perkin Elmer 5 µL” (Perkin Elmer, Waltham, MA, USA). The chromatographic separation was performed on a HP-FFAP capillary column (50 m × 0.32 mm × 0.50 µm) with poly(ethylene glycol) modified by nitroterephthalic acid (CAS number 19091F-115, Agilent Technologies, USA). The initial temperature of the column was 100 °C for 1 min, then increased to 200 °C at a rate of 10 °C /min, which was maintained for 6 min. The total run time was 17 min. The injection temperature was 230 °C, and the detector temperature was −220 °C. Nitrogen was used as a carrier gas in a constant flow mode, and the flow rate was 1.3 mL/min. The make-up gas (N2) was at 20 mL/min. The flow rate of H2 and air were 45 mL/min and 450 mL/min, respectively. For analysis, 2 μL of the sample solution was injected in split mode at a ratio of 20:1. The amount of SCFAs in the sample solution was determined using the software “TotalChrom ver. 6.3.2” (Perkin Elmer, Waltham, MA, USA) by the external-standard method.

### 4.4. Statistical Analysis

To assess the differences between the alpha and beta diversity, as well as the taxonomic and functional composition of the gut microbiota between time points within the same treatment group, a Wilcoxon signed-rank test with Benjamini–Hochberg correction for multiple comparisons was employed. The correlation was calculated using Spearman’s method. *p* < 0.05 was considered significant.

## 5. Conclusions

Looking at the data as a whole, *H. pylori* eradication therapy leads to a coordinated set of functional changes rather than isolated pathway alterations. There is a clear reduction in metabolic activity associated with *Actinobacteria*, accompanied by disruption of the cross-feeding networks that normally support SCFA production. While some butyrate-producing taxa recover over time, this recovery is incomplete and uneven. In parallel, *Bacteroides* abundance remains stable and takes on a relatively more prominent metabolic role, contributing to a shift in SCFA balance. Importantly, these changes are still evident four weeks after therapy, indicating that the microbiota does not simply return to its baseline state but instead moves toward a new functional condition.

The fact that some butyrate-producing taxa remained almost unchanged or returned to their initial state, as well as butyric acid level, by four weeks post-eradication therapy may be at least partly related to a protective effect of the butyric acid and inulin supplementation.

To confirm the conclusions, a larger sample size and longer studies are required.

### Limitations of the Study

Increasing the patient sample size in this study design will make it possible to overcome limitations regarding the reliability of the findings, while accounting for the influence of gender, age, medical history, diet, and place of residence.

## Figures and Tables

**Figure 1:**
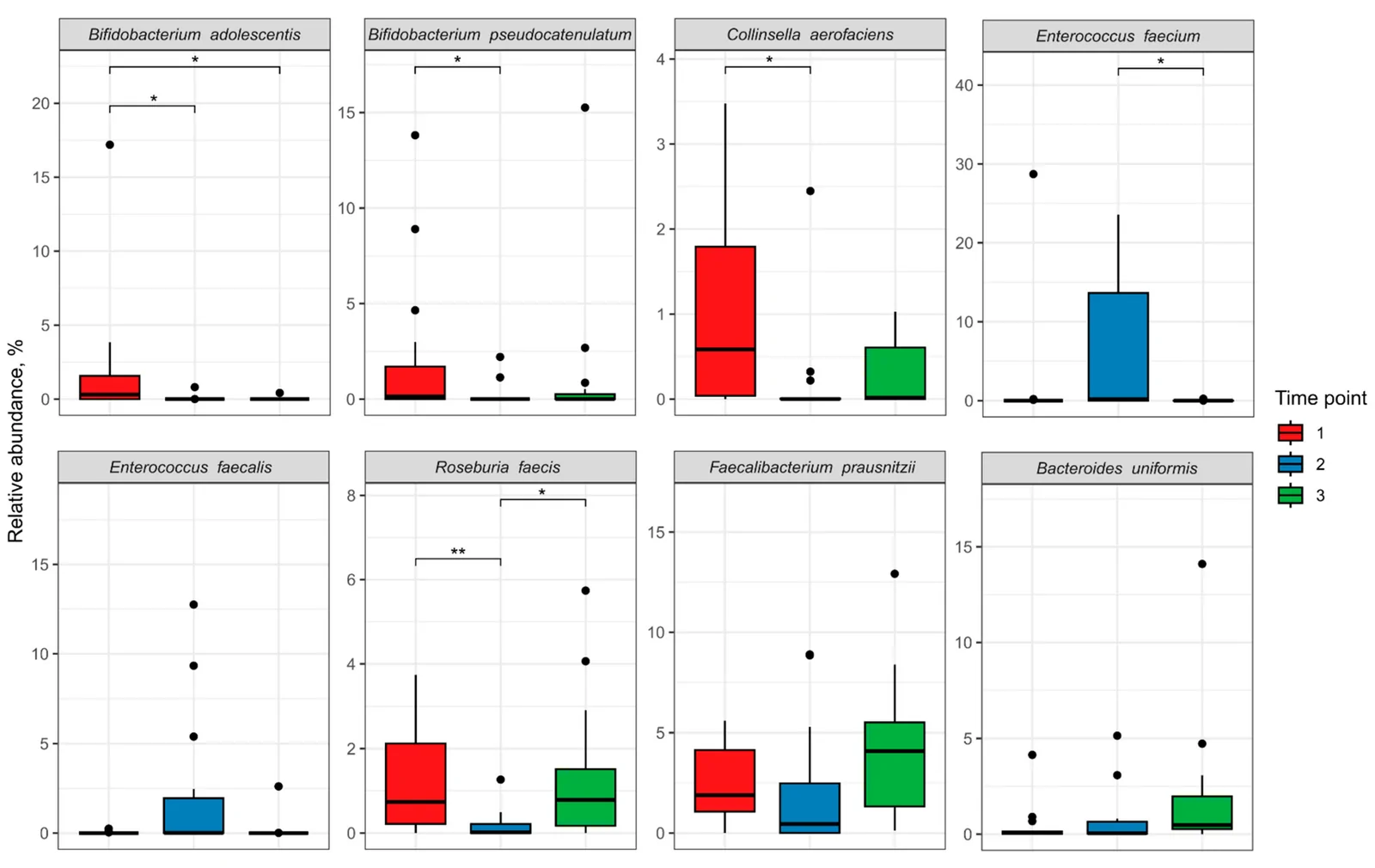
Relative abundance of core bacterial representatives of gut microbiota during *H. pylori* eradication therapy. Point 1—before eradication therapy; Point 2—immediately after eradication therapy; Point 3—4 weeks after the end of eradication therapy. *, *p* < 0.01; **, *p* < 0.05.

**Figure 2:**
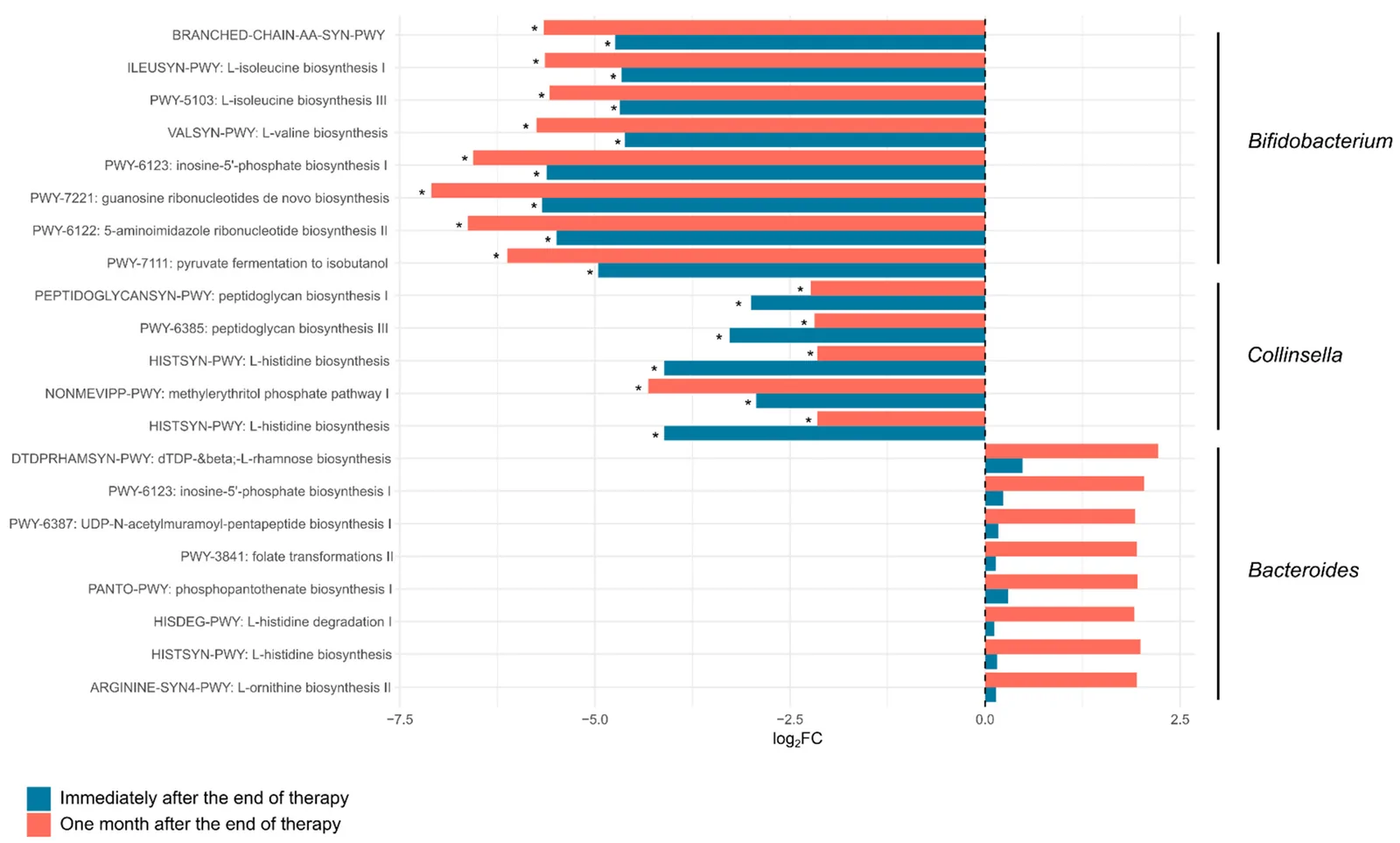
The abundance of pathways, related to *Bifidobacterium*, *Collinsella*, and *Bacteroides*, at Point 2 (immediately after the end of eradication therapy) and Point 3 (four weeks after the end of eradication therapy). *, *p* < 0.05.

**Figure 3:**
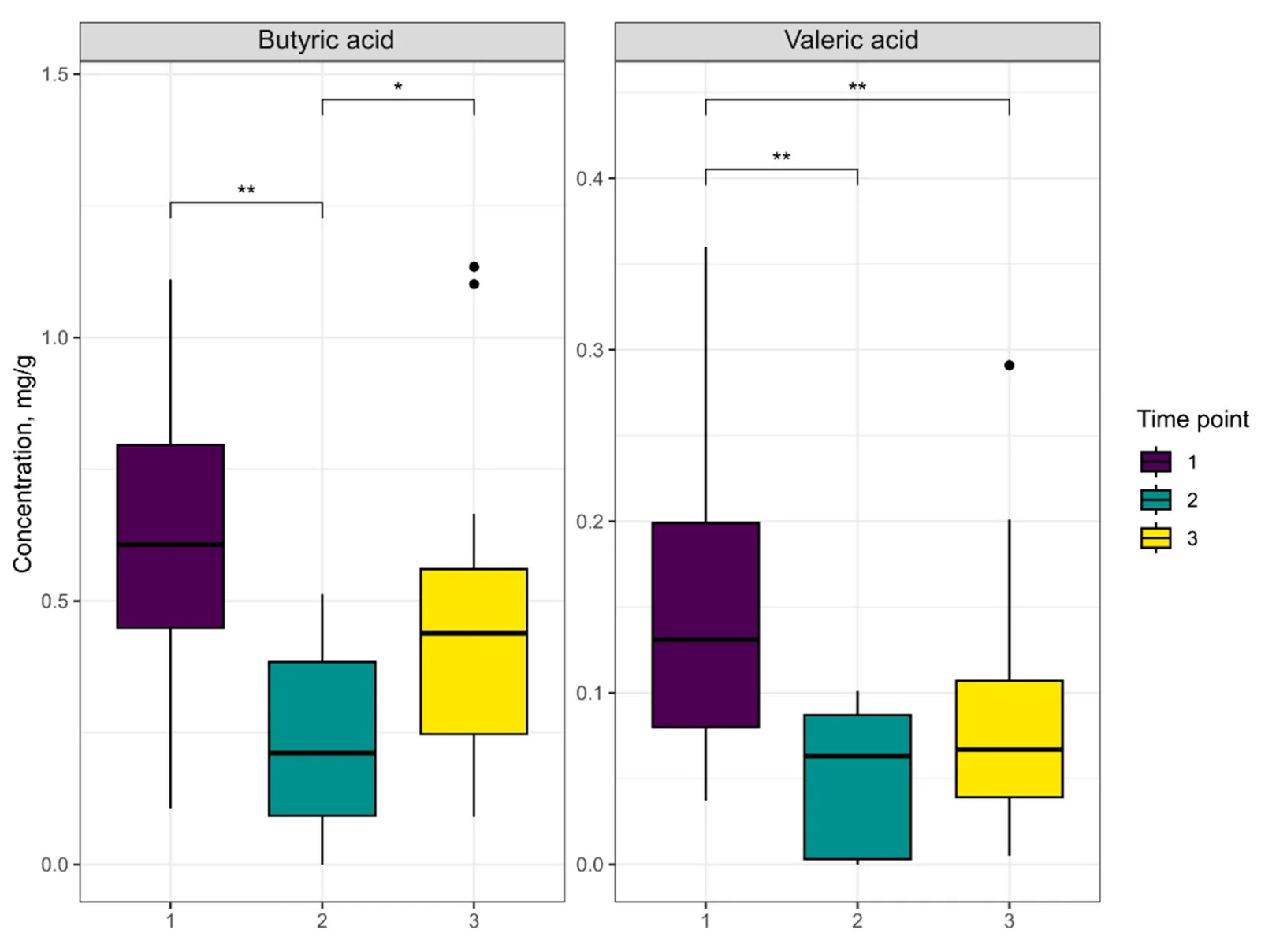
Levels of butyric and valeric acids before (Point 1), immediately after (Point 2), and 4 weeks after eradication therapy (Point 3). *, *p* < 0.05; **, *p* < 0.01.

**Table 1:** Immediate relative abundance changes (baseline (Point 1) vs. post-therapy (Point 2)).

Bacterial Species	P1 Avg. ± SD, %	P2 Avg. ± SD, %	*p*-Value
***Bifidobacterium adolescentis***	1.88 ± 4.39	0.05 ± 0.02	**0.029**
***Bifidobacterium pseudocatenulatum***	2.11 ± 4.09	0.22 ± 0.06	**0.018**
***Collinsella aerofaciens***	1.07 ± 1.17	0.20 ± 0.63	**0.013**
*Enterococcus faecium*	1.93 ± 7.4	6.8 ± 8.57	0.186
*Enterococcus faecalis*	0.03 ± 0.07	0.12 ± 3.96	0.113
***Roseburia faecis***	1.32 ± 1.37	0.18 ± 0.34	**˂0.01**
*Faecalibacterium prausnitzii*	2.46 ± 1.87	2.031 ± 3.14	0.27
*Bacteroides uniformis*	0.43 ± 1.06	0.75 ± 1.45	0.89

Note: P1—baseline (Point 1); P2—immediately after eradication therapy (Point 2); Avg.—average; SD—standard deviation. The *p*-value < 0.05 is highlighted in bold.

**Table 2:** Longitudinal relative abundance changes (baseline (Point 1) vs. post-therapy (Point 3)).

Bacterial Species	Point 1 (Avg. ± SD), %	Point 3 (Avg. ± SD), %	*p*-Value
***Bifidobacterium adolescentis***	**1.88 ± 4.39**	**0.03 ± 0.13**	**0.018**
*Bifidobacterium pseudocatenulatum*	2.11 ± 4.09	1.29 ± 3.93	0.4
*Collinsella aerofaciens*	1.07 ± 1.17	0.28 ± 0.39	0.68
*Bacteroides uniformis*	0.43 ± 1.06	1.98 ± 3.61	0.1
*Roseburia faecis*	1.32 ± 1.36	1.33 ± 0.40	0.85
*Enterococcus faecium*	0.30 ± 1.10	0.02 ± 0.06	0.83
*Enterococcus faecalis*	0.03 ± 0.07	0.18 ± 0.67	1
*Faecalibacterium prausnitzii*	2.45 ± 1.87	4.24 ± 3.37	0.077

Note: P1—baseline (Point 1); P3—4 weeks post-therapy (Point 3); Avg.—average; SD—standard deviation. The *p*-value < 0.05 is highlighted in bold.

**Table 3:** The abundance of branched-chain amino-acid-related metabolic pathways (baseline (Point 1) vs. after eradication therapy (Point 2) vs. post-therapy (Point 3)).

MetaCyc Pathway (*Bifidobacterium*)	P1 Avg. ± SD, cpm	P2 Avg. ± SD, cpm	P3 Avg. ± SD, cpm	*p*-Value (P1 vs. P2)	*p*-Value (P1 vs. P3)
BRANCHED-CHAIN-AA-SYN-PWY	11.89 ± 23.24	0.44 ± 1.72	0.23 ± 0.91	**0.027**	**0.027**
ILEUSYN-PWY (L-isoleucine I)	11.24 ± 22.06	0.44 ± 1.72	0.22 ± 0.87	**0.027**	**0.027**
PWY-5103 (L-isoleucine II)	10.25 ± 19.78	0.39 ± 1.54	0.21 ± 0.82	**0.027**	**0.027**
VALSYN-PWY (L-valine)	12.26 ± 24.47	0.5 ± 1.93	0.22 ± 0.88	**0.037**	**0.027**

Note: P1—baseline (Point 1); P2—immediately after eradication therapy (Point 2); P3—4 weeks post-therapy (Point 3); Avg.—average; SD—standard deviation. The *p*-value < 0.05 is highlighted in bold.

**Table 4:** The abundance of nucleotide biosynthesis and salvage metabolic pathways (baseline (Point 1) vs. after eradication therapy (Point 2) vs. post-therapy (Point 3)).

MetaCyc Pathway (*Bifidobacterium*)	P1 Avg. ± SD, cpm	P2 Avg. ± SD, cpm	P3 Avg. ± SD, cpm	*p*-Value (P1 vs. P2)	*p*-Value (P1 vs. P3)
PWY-6123: inosine-5′-phosphate biosynthesis I	10.75 ± 26.36	0.21 ± 0.84	0.11 ± 0.44	**0.027**	**0.027**
PWY-7221: guanosine ribonucleotides de novo biosynthesis	15.32 ± 38.74	0.29 ± 1.15	0.11 ± 0.43	**0.045**	**0.043**
PWY-6122: 5-aminoimidazole ribonucleotide biosynthesis II	10.81 ± 26.71	0.24 ± 0.93	0.11 ± 0.42	**0.037**	**0.027**

Note: P1—baseline (Point 1); P2—immediately after eradication therapy (Point 2); P3—4 weeks post-therapy (Point 3); Avg.—average; SD—standard deviation. The *p*-value < 0.05 is highlighted in bold.

**Table 5:** The abundance of energy metabolism and fermentation shunts metabolic pathways (baseline (Point 1) vs. after eradication therapy (Point 2) vs. post-therapy (Point 3)).

MetaCyc 1.	P1 Avg. ± SD, cpm	P2 Avg. ± SD, cpm	P3 Avg. ± SD, cpm	*p*-Value (P1 vs. P2)	*p*-Value (P1 vs. P3)
PWY-7111: pyruvate fermentation to isobutanol (engineered)	11.09 ± 24.85	0.35 ± 1.38	0.15 ± 0.61	**0.027**	**0.027**

Note: P1—baseline (Point 1); P2—immediately after eradication therapy (Point 2); P3—4 weeks post-therapy (Point 3); Avg.—average; SD—standard deviation. The *p*-value < 0.05 is highlighted in bold.

**Table 6:** The abundance of peptidoglycan biosynthesis and immunomodulation-related pathways (baseline (Point 1) vs. after eradication therapy (Point 2) vs. post-therapy (Point 3)).

MetaCyc Pathway (*Collinsella*)	P1 Avg. ± SD, cpm	P2 Avg. ± SD, cpm	P3 Avg. ± SD, cpm	*p*-Value (P1 vs. P2)	*p*-Value (P1 vs. P3)
PEPTIDOGLYCANSYN-PWY: peptidoglycan biosynthesis I	10.31 ± 11.63	1.29 ± 5.005	2.19 ± 3.907	**0.024**	**0.048**
PWY-6385: peptidoglycan biosynthesis III	10.06 ± 11.40	1.03 ± 4.03	2.21 ± 3.92	**0.024**	**0.048**
HISTSYN-PWY: L-histidine biosynthesis	6.25 ± 7.2	0.36 ± 1.4	1.41 ± 3.13	**0.039**	**0.048**

Note: P1—baseline (Point 1); P2—immediately after eradication therapy (Point 2); P3—4 weeks post-therapy (Point 3); Avg.—average; SD—standard deviation. The *p*-value < 0.05 is highlighted in bold.

**Table 7:** The abundance of terpenoid biosynthesis and the methylerythritol phosphate pathway (baseline (Point 1) vs. after eradication therapy (Point 2) vs. post-therapy (Point 3)).

MetaCyc Pathway (*Collinsella*)	P1 Avg. ± SD, cpm	P2 Avg. ± SD, cpm	P3 Avg. ± SD, cpm	*p*-Value (1 vs. 2)	*p*-Value (1 vs. 3)
NONMEVIPP-PWY: methylerythritol phosphate pathway I	7.05 ± 7.87	0.92 ± 3.58	0.35 ± 1.37	**0.031**	**0.031**

Note: P1—baseline (Point 1); P2—immediately after eradication therapy (Point 2); P3—4 weeks post-therapy (Point 3); Avg.—average; SD—standard deviation. The *p*-value < 0.05 is highlighted in bold.

**Table 8:** The abundance of carbohydrate and sugar-nucleotide pathways (baseline (Point 1) vs. after eradication therapy (Point 2) vs. post-therapy (Point 3)).

MetaCyc Pathway (*B. uniformis*)	P1 Avg. ± SD, cpm	P2 Avg. ± SD, cpm	P3 Avg. ± SD, cpm	*p*-Value (P1 vs. P2)	*p*-Value (P1 vs. P3)
DTDPRHAMSYN-PWY (dTDP-L-rhamnose)	3.36 ± 9.29	4.69 ± 8.91	15.66 ± 25.06	0.73	0.050
PWY-6123 (Inosine-5′-P II)	2.42 ± 6.48	2.85 ± 5.54	9.93 ± 15.55	0.906	0.098
PWY-6387 (UDP-N-acetylmuramoyl-pentapeptide)	2.31 ± 6.29	2.6 ± 5.06	8.76 ± 14.23	1	0.124

Note: P1—baseline (Point 1); P2—immediately after eradication therapy (Point 2); P3—4 weeks after the end of eradication therapy (Point 3); Avg.—average; SD—standard deviation.

**Table 9:** The abundance of folate and vitamin metabolism-related pathways (baseline (Point 1) vs. after eradication therapy (Point 2) vs. post-therapy (Point 3)).

MetaCyc Pathway (*B. uniformis*)	P1 Avg. ± SD, cpm	P2 Avg. ± SD, cpm	P3 Avg. ± SD, cpm	*p*-Value (P1 vs. P2)	*p*-Value (P1 vs. P3)
PWY-3841: folate transformations II	2.78 ± 7.55	3.06 ± 5.79	10.71 ± 17.05	1	0.098
PANTO-PWY: phosphopantothenate biosynthesis	2.41 ± 6.53	2.95 ± 5.71	9.33 ± 14.74	0.81	0.098

Note: P1—baseline (Point 1); P2—immediately after eradication therapy (Point 2); P3—4 weeks post-therapy (Point 3); Avg.—average; SD—standard deviation.

**Table 10:** The abundance of histidine and arginine biosynthesis pathways (baseline (Point 1) vs. after eradication therapy (Point 2) vs. post-therapy (Point 3)).

MetaCyc Pathway	Taxon	P1 Avg. ± SD, cpm	P2 Avg. ± SD, cpm	P3 Avg. ± SD, cpm	*p*-Value (P1 vs. P2)	*p*-Value (P1 vs. P3)	Direction of Changes
HISDEG-PWY (His deg)	*B. uniformis*	2.34 ± 6.2	2.54 ± 4.98	8.82 ± 14.1	1	0.098	↑
HISTSYN-PWY: L-histidine biosynthesis	*B. uniformis*	2.72 ± 7.17	3.03 ± 6.07	10.84 ± 17.29	1	0.124	↑
HISTSYN-PWY: L-histidine biosynthesis	*C. aerofaciens*	6.25 ± 7.21	0.36 ± 1.40	1.41 ± 3.13	0.039	**0.048**	↓
ARGININE-SYN4-PWY	*B. uniformis*	2.19 ± 5.87	2.42 ± 4.53	8.45 ± 13.23	0.83	0.098	↑

Note: P1—baseline (Point 1); P2—immediately after eradication therapy (Point 2); P3—4 weeks post-therapy (Point 3); Avg.—average; SD—standard deviation. ↑—increased abundance of genes in the pathway; ↓—decreased abundance of genes in the pathway. The *p*-value < 0.05 is highlighted in bold.

**Table 11:** Correlation of butyric and valeric acids with bacterial species.

	Butyric Acid		Valeric Acid	
	Spearman’s Coef.	*p*-Value	Spearman’s Coef.	*p*-Value
*Bifidobacterium pseudocatenulatum*	**0.32**	**0.033**	0.13	0.37
*Enterococcus faecium*	−0.42	**0.006**	−0.24	0.12
*Faecalibacterium prausnitzii*	0.36	**0.017**	0.37	**0.013**
*Bacteroides uniformis*	−0.25	0.096	−0.39	**0.009**

Note: The *p*-value < 0.05 is highlighted in bold.

## Data Availability

The original data presented in the study are openly available at: https://www.ncbi.nlm.nih.gov/bioproject/PRJNA1063508 (accessed on 1 August 2026).
